# Supplementary material for: Immobilization Systems of Antimicrobial Peptide Ib−M1 in Polymeric Nanoparticles Based on Alginate and Chitosan
Source: Polymers (Basel). 2022 Aug 2;14(15):3149. doi: 10.3390/polym14153149 (PMC9370884; doi:10.3390/polym14153149)
Supplement: Supplementary file 1 [file polymers-14-03149-s001.zip › polymers-1809985-supplementary.pdf]

polymers

MDPI

*Supplementary*

# Immobilization Systems of Antimicrobial Peptide Ib–M1 in Polymeric Nanoparticles Based on Alginate and Chitosan

**Carlos Enrique Osorio-Alvarado [1,*], Jose Luis Ropero-Vega [1], Ana Elvira Farfán-García [2] and Johanna Marcela Flórez-Castillo [1,*]**

[1] Universidad de Santander, Facultad de Ciencias Naturales, Ciencias Básicas y Aplicadas para la Sostenibilidad-CIBAS, Calle 70 No. 55-210, Bucaramanga 680003, Colombia; jose.ropero@udes.edu.co
[2] Universidad de Santander, Facultad de Ciencias Médicas y de la Salud, Instituto de Investigación Masira, Calle 70 No. 55-210, Bucaramanga 680003, Colombia; afarfan@udes.edu.co
* Correspondence: c.osorio1202@gmail.com (C.E.O.-A.); johanna.florez@udes.edu.co (J.M.F.-C.); Tel.: +57-7606-516500 (ext. 1665) (J.M.F.-C.)

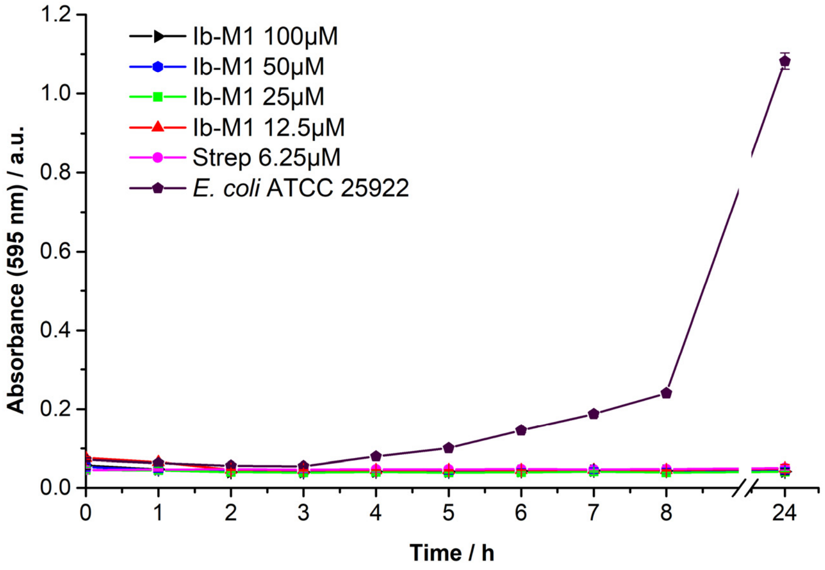


**Figure S1. A.** Growth kinetics of *E. coli* ATTCC 25922 in the presence of peptide Ib-M1 at concentrations of 100 μM (►), 50 μM (⬢), 25 μM (■), 12.5 μM (▲), streptomycin used as reference antibiotic 6.25 μM (●), *E. coli* as growth control (⬟).

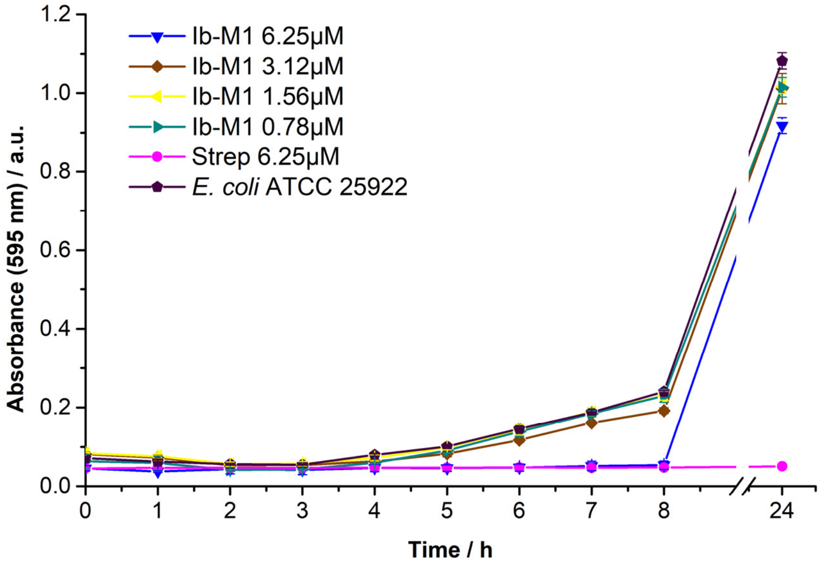


**Figure S1. B.** Growth kinetics of *E. coli* ATTCC 25922 in the presence of peptide Ib-M1 at concentrations 6.25µM (▼), 3.12 µM (◆), 1.56 µM (◂), 0.78 µM (▸), streptomycin used as reference antibiotic 6.25 µM (●), *E. coli* as growth control (⬟).
